# Automatic healthy liver segmentation for holmium-166 radioembolization dosimetry

**DOI:** 10.1186/s13550-023-00996-1

**Published:** 2023-07-15

**Authors:** Martina Stella, Rob van Rooij, Marnix G. E. H. Lam, Hugo W. A. M. de Jong, Arthur J. A. T. Braat

**Affiliations:** Department of Radiology and Nuclear Medicine, UMC Utrecht, Heidelberglaan 100, 3584 CX Utrecht, The Netherlands

**Keywords:** Radioembolization, ^166^Holmium, ^99m^Technetium, Segmentation, Dosimetry

## Abstract

**Background:**

For safe and effective holmium-166 (^166^Ho) liver radioembolization, dosimetry is crucial and requires accurate healthy liver definition. The current clinical standard relies on manual segmentation and registration of a separately acquired contrast enhanced CT (CECT), a prone-to-error and time-consuming task. An alternative is offered by simultaneous imaging of ^166^Ho and technetium-99m stannous–phytate accumulating in healthy liver cells (^166^Ho–^99m^Tc dual-isotope protocol). This study compares healthy liver segmentation performed with an automatic method using ^99m^Tc images derived from a ^166^Ho–^99m^Tc dual-isotope acquisition to the manual segmentation, focusing on healthy liver dosimetry and corresponding hepatotoxicity. Data from the prospective HEPAR PLuS study were used. Automatic healthy liver segmentation was obtained by thresholding the ^99m^Tc image (no registration step required). Manual segmentation was performed on CECT and then manually registered to the SPECT/CT and subsequently to the corresponding ^166^Ho SPECT to compute absorbed dose in healthy liver.

**Results:**

Thirty-one patients (66 procedures) were assessed. Manual segmentation and registration took a median of 30 min per patient, while automatic segmentation was instantaneous. Mean ± standard deviation of healthy liver absorbed dose was 18 ± 7 Gy and 20 ± 8 Gy for manual and automatic segmentations, respectively. Mean difference ± coefficient of reproducibility between healthy liver absorbed doses using the automatic versus manual segmentation was 2 ± 6 Gy. No correlation was found between mean absorbed dose in the healthy liver and hepatotoxicity.

**Conclusions:**

^166^Ho–^99m^Tc dual-isotope protocol can automatically segment the healthy liver without hampering the ^166^Ho dosimetry assessment.

*Trial registration*: ClinicalTrials.gov, NCT02067988. Registered 20 February 2014. https://clinicaltrials.gov/ct2/show/NCT02067988

**Supplementary Information:**

The online version contains supplementary material available at 10.1186/s13550-023-00996-1.

## Introduction

Radioembolization has been used for many years for treatment of non-operable primary and/or metastatic liver lesions. Among commercially available devices, holmium-166 (^166^Ho) microspheres (QuiremSpheres™/QuiremScout™, Quirem Medical BV) allow the use of the same particles for dosimetric assessment during treatment planning and for post-treatment evaluation. In line with conventional radiation oncology, dosimetry plays a key role during these phases.

For hepatic radioembolization, dosimetry is calculated using a partition model which assumes that distinct volumes of interest (VOIs) correspond to different sets of compartments, mainly: healthy liver and tumors [1]. Current clinical practice for ^166^Ho radioembolization relies on manual segmentation of these compartments on a previously acquired contrast enhanced CT (CECT) or MRI, and its subsequent registration to the low-dose CT (LDCT) accompanying the SPECT. These two tasks (manual segmentation and registration) present multiple drawbacks, primarily time-consuming and prone to error. Additionally, the manual segmentation and registration introduce an inter-observer variability [2]. These drawbacks might prevent systematic implementation of personalized dosimetry into clinical practice and introduces variability in clinical studies focused on dosimetry. Ultimately this may hinder determination of dose–response relationships and dose–toxicity relationships, and potential treatment outcome prediction.

An alternative to manual segmentation and subsequent manual registration is provided in a protocol based on simultaneous imaging of ^166^Ho, simulating the distribution of microspheres [3] in tumors and healthy liver tissue, and technetium-99m (^99m^Tc) stannous–phytate, accumulating in Kupffer cells located only in healthy tissue (^166^Ho–^99m^Tc dual isotope) [4]. As Kupffer cells are absent in tumorous tissue, this allows a differentiation between tumorous and healthy liver tissue, enabling physiological healthy liver tissue delineation based on [^99m^Tc]-stannous–phytate uptake [5]. This protocol was applied in a clinical study (HEPAR PLuS) and feasibility of tumor dosimetry on ^166^Ho images deriving from this dual-isotope acquisition was previously demonstrated [6].

The aim of this study is to compare dosimetric assessments by manual segmentation (current standard clinical practice) versus the proposed automatically defined healthy liver VOI using the ^166^Ho–^99m^Tc dual-isotope protocol. Furthermore, based on the automatic healthy liver segmentations and the corresponding dosimetry results, a dose–hepatotoxicity relation will be investigated.

## Materials and methods

### Data population

For all SPECT/CT acquisitions used in this study, informed consent was obtained as part of the HEPAR PLuS study (NCT02067988) [7, 8]. The study has been approved by the institutional review board, and all subjects signed an informed consent form. This study included 31 patients with liver metastases of neuroendocrine tumors (NET), 33 pre-treatment procedures and 38 therapeutic treatments. Baseline characteristics are presented in Table 1.

**Table 1:** Baseline characteristics of patients with neuroendocrine liver metastases treated in the HEPAR PLuS trial

	*N* (%)
Number of patients	31
Age—median (IQR)^a^	65.1 (57.6–70.2)
Gender	
Male (%)	23 (74.2%)
Female (%)	8 (25.8%)
Origin of tumor	
Pancreas	10 (32.3%)
Small intestine	8 (25.8%)
Colorectal	4 (12.9%)
Lung	3 (9.7%)
Unknown primary	6 (19.4%)
ECOG	
0	17 (54.8%)
1	13 (41.9%)
2	1 (3.2%)
WHO grade	
1	12
2	19
Tumor^b^ burden in %—median (IQR)	6.9% (3.1–22.5%)
Procedures	70
Pre-treatment	32
Post-treatment	38
Holmium-166 activity in GBq—median (IQR)	5.4 (3.6–8.0)
Technetium-99 m activity in MBq—median (IQR)	52 (50–53)

*IQR* Interquartile range, *ECOG* Eastern Cooperative Oncology Group, *WHO* World Health Organization

^a^At first treatment

^b^It refers to tumor computed on the CECT acquired at the baseline

According to study protocol, for each of the procedures, a SPECT/CT image was acquired after additional activity injection of [^99m^Tc]-stannous–phytate. For the dataset under consideration, clinical outcomes and lung shunt were previously reported [6, 7].

### Images acquisition and reconstruction

All patients were scanned on a Symbia T16 dual-head SPECT/CT scanner (Siemens), using a medium-energy low-penetration collimator, on a 128 × 128 matrix (pixel spacing, 4.8 × 4.8 mm), with 120 angles (15 s per projection) over a non-circular 360° orbit. An energy window centered at 81 keV (15% width), together with an additional energy window centered at 118 keV (12% width) to correct the ^166^Ho photopeak data for downscatter using a window-based scatter correction were used [9]. ^99m^Tc was imaged using a 140 keV (15% width) energy window, with an upper scatter window at 170 keV (12% width) to correct for ^166^Ho downscatter. ^99m^Tc was reconstructed with triple-energy-window scatter correction, using both 118 keV and 170 keV scatter windows. SPECT/CT was acquired following the ^166^Ho radioembolization procedure when activity had decayed to approx. 250 MBq, to avoid dead time effects. Five minutes before start of the SPECT scan, ~ 50 MBq of [^99m^Tc]-stannous–phytate was intravenously injected, to perform the simultaneous ^166^Ho–^99m^Tc dual-isotope acquisition. Reconstruction parameters for both ^166^Ho and ^99m^Tc SPECT were previously analyzed in a phantom study and used in this study [9]. Data were retrospectively assessed to compute the dose in the healthy liver VOI. To this purpose, two approaches were used: manual segmentation and manual rigid registration of CECT on top of SPECT, currently used in clinical practice, and the automatic segmentation on intrinsically registered ^99m^Tc SPECT using a threshold-based method.

### Manual segmentation

For each patient, healthy liver and tumor VOI were manually segmented by an experienced nuclear medicine physician (AJATB) using commercially available software dedicated to ^166^Ho radioembolization (Q-Suite™ 2.1, Quirem Medical BV). Delineation of VOI was performed on pre-treatment CECT. Subsequently, the CECT was rigidly registered on the SPECT acquired after the ^166^Ho administration procedure through the corresponding LDCT intrinsically registered to the SPECT. Healthy liver VOI was defined by subtracting the manually segmented tumor VOI from total liver volume VOI. Additionally, necrotic tissue was manually segmented as well, to be excluded from healthy liver VOI. Only tumors with a diameter greater than 1 cm were included in the VOI processing. Manual segmentation time for each patient was recorded.

### Automatic segmentation

**Fig. 1:**
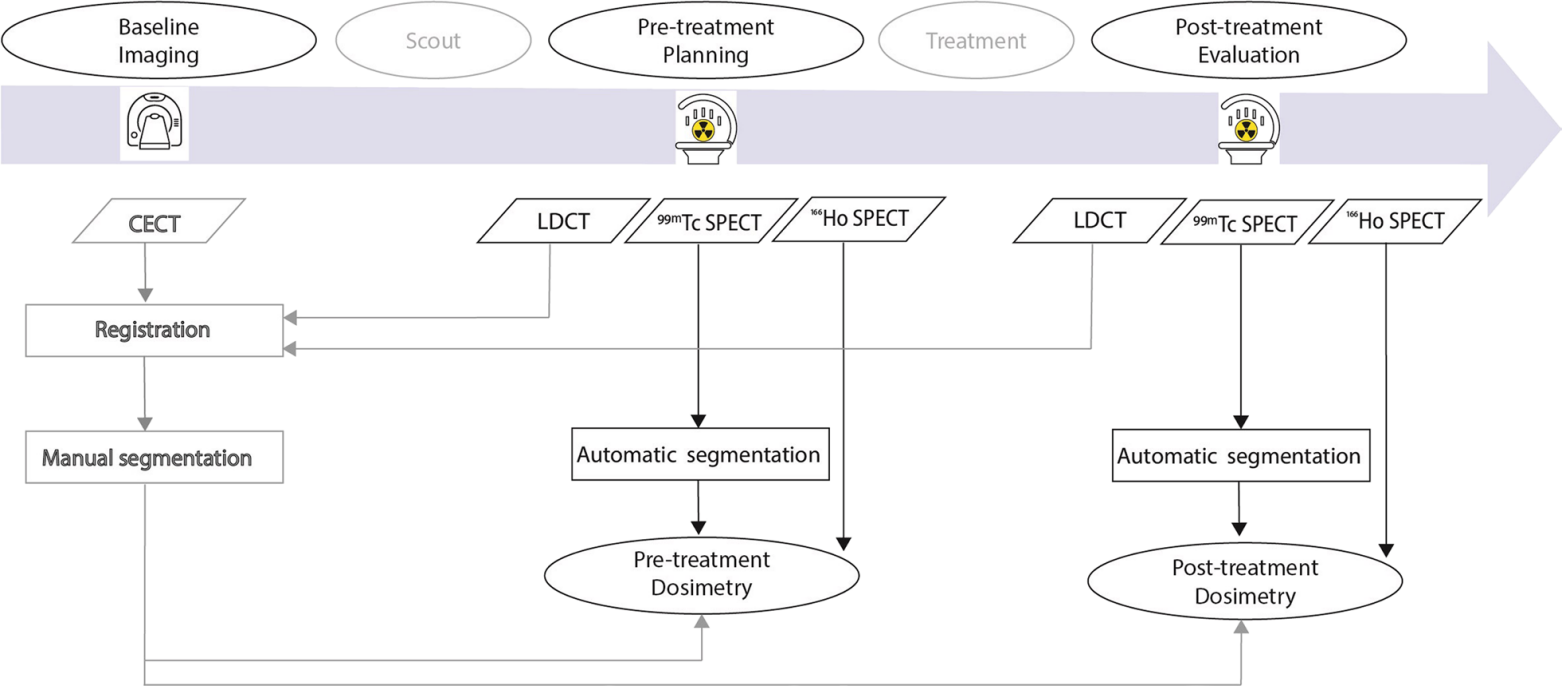
Schematic workflow representing the comparison between manual (left) and automatic (middle and right) segmentation with respect to the dosimetry purpose. The methods have been integrated with corresponding inputs and their timeline within the ^166^Ho radioembolization workflow (upper blue arrow)

Since [^99m^Tc]-stannous–phytate accumulates also in the spleen, a preliminary procedure was applied to remove the spleen from the ^99m^Tc reconstructions. Subsequently, automatic healthy liver segmentation was performed on the ^99m^Tc reconstruction using a threshold method previously assessed in a phantom study [9]. First, the maximum value in a smoothened version of the ^99m^Tc image is determined. Then, the original ^99m^Tc image is thresholded to 40% of this maximum to produce the segmentation. Figure 1 illustrates the manual and automatic segmentation methods within the ^166^Ho radioembolization workflow.

### Dosimetry

To estimate dose in healthy liver VOI, all ^166^Ho images were converted to MBq/ml using administered therapeutic activity, on the assumption that all activity was in the SPECT field of view. ^166^Ho images were scaled considering only counts within the patient–body mask, automatically delineated on LDCT acquired together with SPECT. Since some patients received a partial liver treatment, ^166^Ho pre-treatment images were scaled either using the partial treatment activity or the sum of the activities administered combining the complementary treatments. For all VOIs, dose was computed as:

$${\text{Dose}}\left[ {{\text{Gy}}} \right] = 15.87\left[ {\frac{{{\text{mJ}}}}{{{\text{MBq}}}}} \right]\frac{{ {\text{Activity}}\;{\text{ concentration}}_{{{\text{VOI}}}} \left[ {\text{MBq/ml}} \right]}}{{{\text{VOI}}\;{\text{ density}}\;\left[ {\text{g/ml}} \right]}}$$

where 15.87 mJ/MBq represents the deposited energy due to *β* decay of 1 MBq ^166^Ho. For the liver, a soft tissue density of 1.06 g/cm^3^ was applied, assuming a homogenous organ density value, constant among patients.

### Segmentation evaluation

Healthy liver segmentations obtained using the manual and automatic approach were compared considering the resulting healthy liver volume difference and using two overlapping indices: the Sørensen–Dice coefficient (SDC) and the Hausdorff distance (HD). Since mean absorbed dose within the considered VOI was deemed as a more representative clinical metric, a Bland–Altman plot was used to compare resulting mean absorbed dose computed in the healthy liver manually and automatically segmented. Pearson’s test was used to assess the correlation. As there is no current standard for dose–volume histogram (DVH) reporting, minimum dose to 70% of the volume (*D*_70_) and volume receiving at least 50 Gy (*V*_50_) were used to compare the two segmentations methods [10]. In each comparative analysis, manual segmentation was considered as reference standard.

Hepatotoxicity was assessed at baseline and up to 12 months, every 3 months, according to the five point standardized scale of hepatotoxicity after radioembolization proposed by Braat et al. [11], and a score of ≥ 3 was considered significant. To assess the dose–hepatotoxicity relation for patients who underwent multiple treatments, mean absorbed doses in the healthy liver resulting from all the treatments received up to the time point under investigation were summed. Dose–hepatotoxicity relation was investigated considering the worst grade hepatotoxicity during the follow-up and cumulative absorbed dose in the healthy liver received by that time point.

To check the statistical difference between resulting doses computed on manual and automatic segmentation (null hypothesis is no difference), a two-sided paired t test (at *α* = 0.05) was performed.

## Results

For 31 subjects and a total of 66 procedures (29 pre- and 37 post-treatment), ^166^Ho–^99m^Tc dual-isotope acquisition was performed and included for segmentation assessment. One patient died within 3 months after ^166^Ho radioembolization because of a hypoglycemic crisis caused by an overproducing insulinoma. For another patient who underwent two radioembolization treatments, ^166^Ho–^99m^Tc dual-isotope acquisition was available for only one of the two treatments. These patients were included in the segmentation method comparison, but were excluded in the dose–hepatotoxicity correlation analysis. This resulted in a total of 29 subjects (30 treatments) assessed for hepatotoxicity during follow-up.

Manual segmentation and registration of CECT onto SPECT took a median (IQR) of 30 (15) minutes per patient, while automatic segmentation was instantaneous. Median (IQR) difference between healthy liver volume automatically and manually segmented was 171 mL (341 mL), resulting in a statistically significant difference (*p* < 0.01). Median (IQR) SDC and HD were 0.8 (0.12) and 8.6 cm (1.4 cm), respectively.

**Fig. 2:**
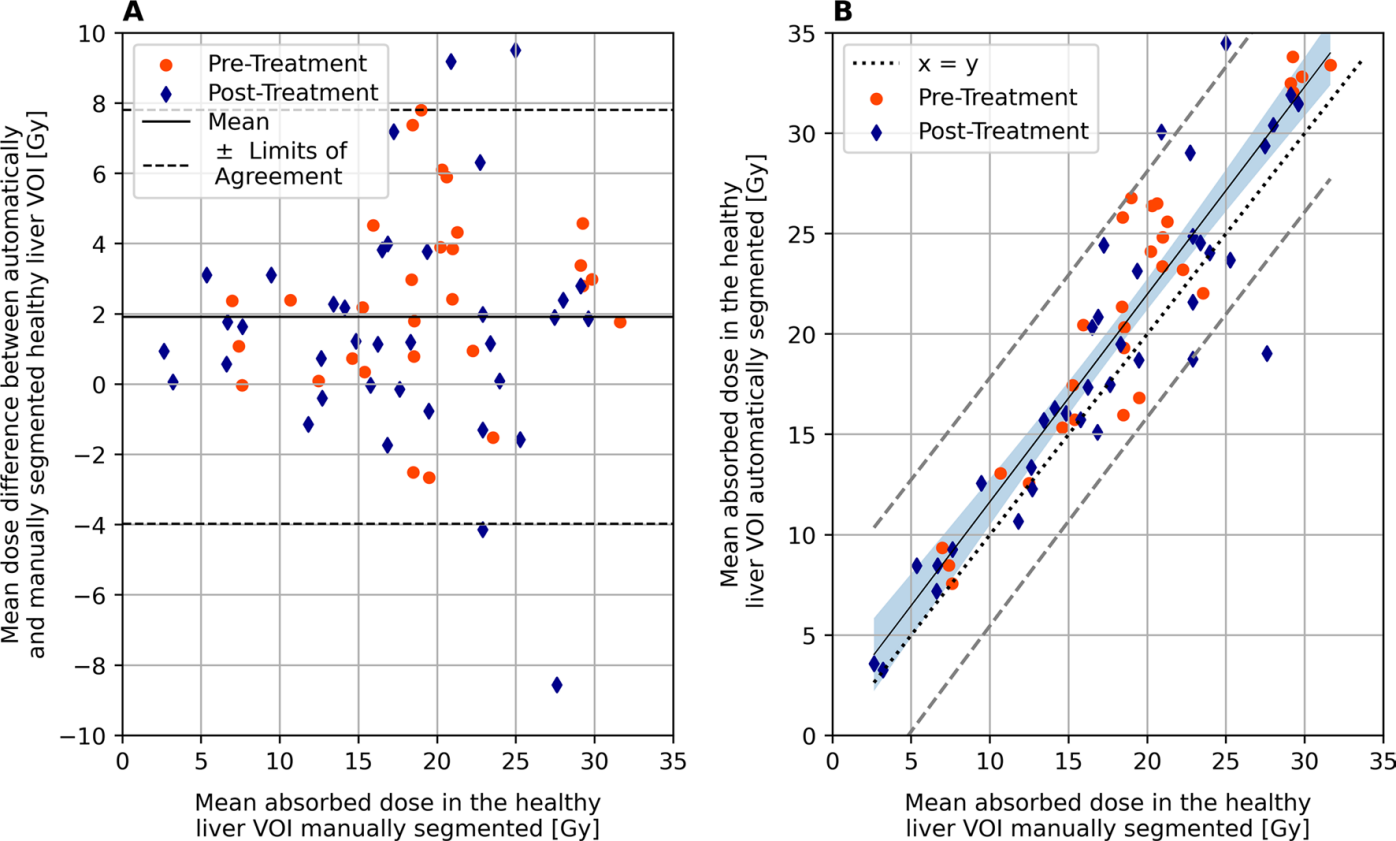
**A** Bland–Altman plot on the difference between mean absorbed dose. Mean of the difference is depicted by the black solid line, while black dashed lines show limits of agreement. **B** linear correlation plot between manual and automatic segmentation of the healthy liver VOI with respect to the mean absorbed dose. The solid line depicts linear regression (*R*^2^ = 0.92), while the dashed lines indicate the ± 95% confidence intervals. Dotted line represents the *x* = *y* line. ^166^Ho pre-treatment images were scaled considering the therapeutic activity

Mean ± standard deviation of health liver absorbed dose was 18 ± 7 Gy and 20 ± 8 Gy for manual and automatic segmentations, respectively. Bland–Altman plot and correlation between dose computed in the healthy liver manually and automatically segmented are shown in Fig. 2. Mean difference ± coefficient of reproducibility between healthy liver absorbed doses using the automatic segmentation versus manual segmentation was 2 ± 6 Gy (Fig. 2**A**), resulting in limit of agreement of 8 Gy and − 4 Gy. A statistically significant difference in mean healthy liver absorbed doses was found between the two methods (*p* < 0.01), with the dose computed by automated segmentation, on average, 2 Gy higher. The linear correlation between healthy liver dose computed using these two methods, assessed using Pearson’s correlation coefficient (Fig. 2**AB**), was 0.92.

Mean ± standard deviation of *D*_70_ was 10 ± 6 Gy and 11 ± 6 Gy for manual and automatic segmentations, respectively. A Bland–Altman plot and the correlation between *D*_70_ are shown in Additional file 1: S1. Mean difference ± coefficient of reproducibility between *D*_70_ computed using the automatic segmentation versus manual segmentation was 1 ± 4 Gy (S1**A**). A statistically significant difference in *D*_70_ was found between the two methods, resulting in a mean 1 Gy difference for *D*_70_ by automated segmentation (*p* < 0.01). The linear correlation between *D*_70_ computed using these two methods, assessed using Pearson’s correlation coefficient (S1**B**), was 0.95.

Mean ± standard deviation of *V*_50_ was 3 ± 3% and 4 ± 5% for manual and automatic segmentations, respectively. Bland–Altman plot and correlation between *V*_50_ computed in the healthy liver manually and automatically segmented are shown in Additional file 1: S2. Mean difference ± coefficient of reproducibility between *V*_50_ computed using the automatic segmentation versus manual segmentation was 1 ± 5% (S2**A**). A statistically significant difference in *V*_50_ was found between the two methods (*p* < 0.01), resulting in a mean 1% difference for *V*_50_ by automated segmentation. The linear correlation between *V*_50_ computed using these two methods, assessed using Pearson’s correlation coefficient (S2**B**), was 0.82.

Analysis of hepatotoxicity during the 12 months follow-up after ^166^Ho radioembolization, including the number of subjects per each toxicity grade, together with the corresponding mean absorbed dose in the healthy liver manually and automatically segmented is reported in Table 2.

**Table 2:** Worst hepatotoxicity grade scored according to Braat et al. [11]. Median ± IQR of the absorbed dose in the healthy liver is reported for manual and automatic segmentation

	0	1	2	3	4	5
Number of subjects	1	17	8	1	1	1
Dose manual [Gy]	21	23 ± 7	22 ± 12	19	23	30
Dose automatic [Gy]	30	24 ± 11	21 ± 9	20	22	32

**Fig. 3:**
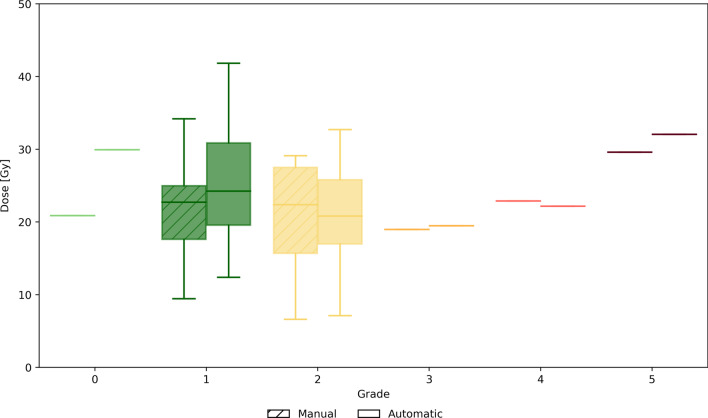
Boxplot representing the healthy liver dose (manually and automatically segmented) for the worst hepatotoxicity grade during follow-up for all 29 patients in the toxicity analysis

Hepatotoxicity with a significant grade (≥ 3) was found in three patients graded 3, 4 and 5 with a corresponding mean absorbed dose in the manually (and automatically) segmented healthy liver of 19 Gy (20 Gy), 23 (22 Gy) and 30 Gy (32 Gy), respectively. No correlation was found between hepatotoxicity and healthy liver dose. A boxplot representing the healthy liver dose (manually and automatically segmented) per each toxicity grade is depicted in Fig. 3.

## Discussion

The ^166^Ho–^99m^Tc dual-isotope protocol offers an automated and reliable alternative to manual segmentation and consequent registration of the healthy liver for future clinical practice. This paves the way to automatic pre-treatment planning and personalized treatment based on healthy liver dosimetry. The ^166^Ho–^99m^Tc dual-isotope protocol can also facilitate rapid post-treatment evaluation, providing insights on dose delivered to the healthy liver and eventually predicting whether significant hepatotoxicity might be encountered during follow-up. Particularly for neuroendocrine tumor patients with long survival times, for whom long-term toxicity is feared [12], healthy liver dose prediction is of even greater importance. The possibility to optimize radioembolization treatment considering the healthy liver dose and corresponding toxicity as limiting factor in order to deliver the maximum tolerable absorbed dose to healthy liver tissue was suggested by Chiesa et al. [13] who investigated this approach for hepatocellular carcinoma patients treated with yttrium-90 glass microspheres.

It is commonly accepted that optimal radioembolization requires accurate dosimetry of both tumor and healthy liver [14, 15]. The mean absorbed dose difference between automatically and manually segmented healthy liver is limited to 2 Gy, which, even though statistically significant, was considered acceptable for healthy liver assessment by experienced nuclear medicine physicians. The same considerations apply to the *D*_70_ and *V*_50_ used to assess the DVH. These results are in line with the results from tumor dosimetry with the same ^166^Ho–^99m^Tc dual-isotope protocol, also 2 Gy overestimation [6]. With these limited differences in mind, ^166^Ho–^99m^Tc dual-isotope protocol seems a viable imaging protocol to enhanced implementation of automated personalized dosimetry in daily clinical practice. However, at this time, the possibility to perform tumor dose assessment is subject to manual segmentation and registration of lesions by physicians. Thus, as for healthy liver, dosimetry would benefit from automatic tumor segmentation. Despite ^166^Ho uptake occurring primarily in tumors (for hypervascular lesions), a minor amount of ^166^Ho is distributed in healthy liver tissue. For this reason, a robust thresholding approach to segment tumors based on the ^166^Ho image alone is challenging to implement. The use of the ^99m^Tc image derived from the ^166^Ho–^99m^Tc dual-isotope protocol for automatic tumors segmentation would rely on automatic identification of cold spots (considered as tumors due to the lack of Kupffer cells present in parenchyma). This approach presents several challenges since the limited SPECT resolution would play a bigger role in identification of small volumes of interest. The resolution will limit detection of small tumors, while miss identification of cold spots as tumors could potentially be benign lesions (e.g., liver cysts). Additionally, to perform automatic tumor dosimetry, the perfused volume during treatment should be identified. While the ^166^Ho image itself provides an indication of the perfused volume, this task is not straightforward (especially for lobar treatments) and brings a further challenge to automatic tumor dosimetry using the ^166^Ho–^99m^Tc dual-isotope protocol.

**Fig. 4:**
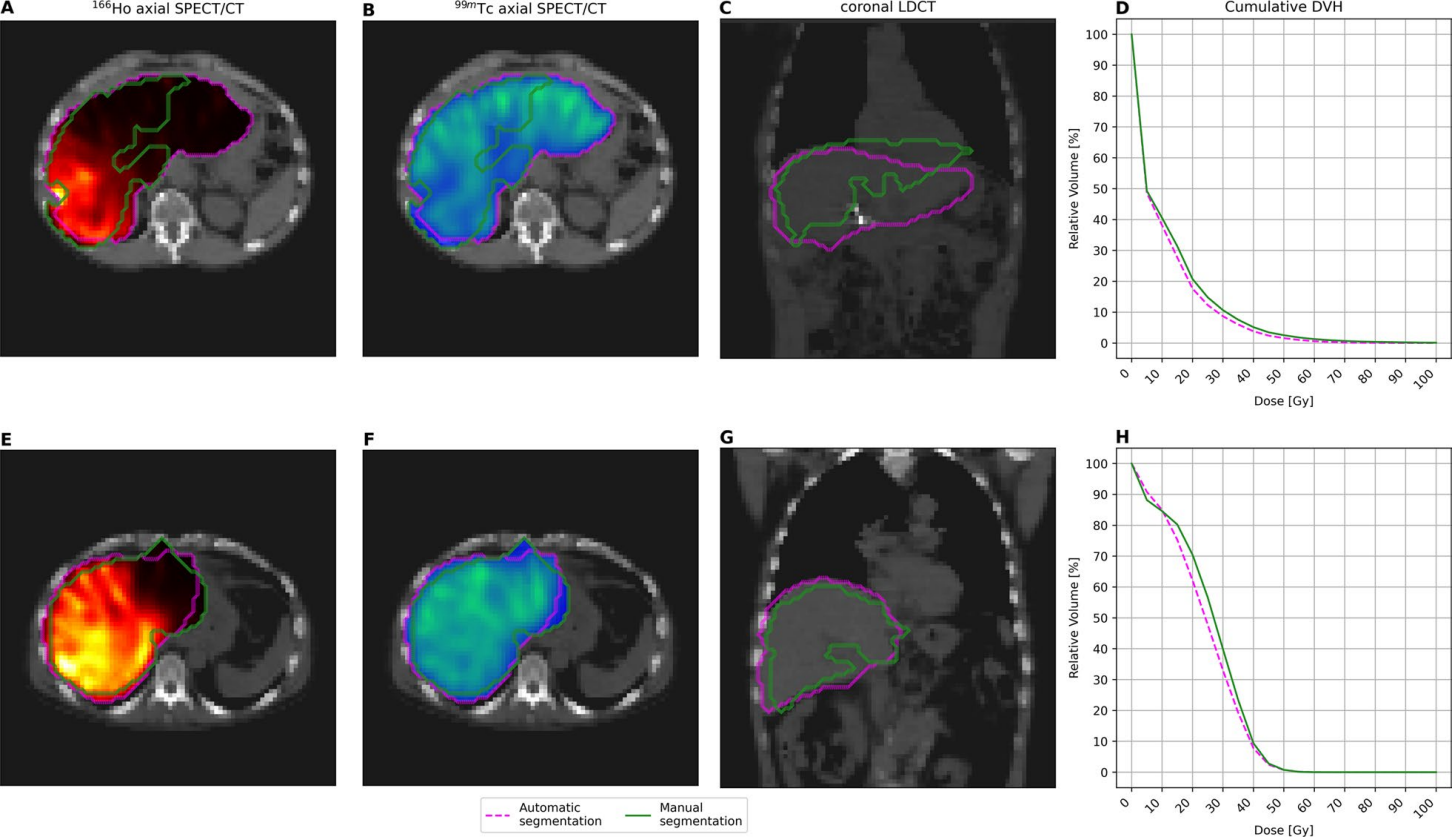
Examples of automatic segmentation outcome. **A**–**D**. A 56-year-old male subject treated with 3850 MBq of ^166^Ho microspheres in the right liver. **E**–**H**. A 65-year-old male subject treated with 4982 MBq of ^166^Ho microspheres in the right liver. Panels **A** and **E**, and **B** and **F**, show the axial view of ^166^Ho and ^99m^Tc SPECT/CT, respectively, while panels **C** and **G** show the coronal view of the LDCT acquired with the SPECT. Manual (solid green) and automatic (dashed magenta) segmentation is depicted. Upper panels (**A**–**C**, Sørensen–Dice coefficient = SDC = 0.62), show a clear discrepancy between segmentations, which is due to non-rigid deformation of liver because of breathing. Segmentations are quite comparable in the bottom panels (**E**–**G**, SDC = 0.84). Panels **D** and **H** show the resulting cumulative dose–volume histogram (DVH). Despite the segmentation differences, the corresponding dose difference is below 2 Gy

Besides saving time (~ 30 min per patient when using the manual segmentation versus instantaneous with this ^166^Ho–^99m^Tc dual-isotope protocol), another straightforward benefit of the ^166^Ho–^99m^Tc dual-isotope protocol is the potential to avoid of inter-observer variability when conducting dosimetry [2]. Several large studies have shown the importance of dosimetry in patient outcome [16, 17], and clinical studies currently enrolling participants implement dosimetric endpoints. Thus, limiting underlying errors (e.g., registration errors and inter-observer variability) will become more important for data generalizability in the future. In this study, some of these variations become apparent, as despite of overlapping indices like SDC and HD, which are easy to implement and useful summary measures of spatial overlap, they poorly outline the considerations assessing the segmentation methods (manual versus automatic) for the purpose of ^166^Ho radioembolization dosimetry. An explanatory example showing how overlapping indices might lead to partial conclusions on the resulting dosimetry is depicted in Fig. 4.

In this study, all patients presented a mean healthy liver absorbed dose (manually or automatically segmented) < 35 Gy (range 3–32 Gy), below the reported acceptable healthy liver dose limits reported for patients with different tumor types treated with radioembolization [13, 18, 19]. However, due to the limited REILD occurrence within this study (1 case) and low incidence of significant hepatotoxicity (1 case), no correlation was found and no strong conclusion can be drawn on maximum tolerable healthy liver dose. This is in line with previous results by Ebbers et al. [20] in which no significant relationship between absorbed dose in treated healthy liver and biochemical toxicity was found when assessing a NET patient population treated with yttrium-90 glass microspheres. To date, no clear dose–toxicity relationships have been described in radioembolization in general; advised thresholds for healthy liver dose are based on limited evidence and often lack validation [21].

Several limitations apply to this study. Primarily, the healthy liver segmentation manually performed on baseline anatomical image and rigidly registered on SPECT is known to be far from ideal (as shown in Fig. 4**A**–**C**); even though it is current clinical practice, the reference standard (‘ground truth’) to which the automatic segmentation outcome was compared is limited. Also, the automatic segmentation method, which relies on a fixed threshold for all patients, can lead to suboptimal segmentation for some patients. [^99m^Tc]-stannous–phytate accumulates in Kupffer cells only, representing healthy liver; however, its uptake is not homogenous and does not provide any information regarding regional liver function. Furthermore, an intrinsic limitation of ^166^Ho–^99m^Tc dual-isotope approach is the limited resolution of SPECT, which does not allow for the identification of small lesions, widely present in NET patients. Additionally, the definition of a clear dose–hepatotoxicity relation is hampered by the limited population size (and number of events) and hepatotoxicity following radioembolization is related to multiple risk factors such as previous liver directed treatments or intra-arterial therapies, tumor burden and toxicity of concomitant medications [11].

^166^Ho–^99m^Tc dual-isotope protocol is a promising and rapid protocol for automated imaging segmentation; however, several challenges still need to be addressed, especially concerning automated tumor dosimetry. A clinical validation study of the ^166^Ho–^99m^Tc dual-isotope protocol, including more patients, should be performed to confirm the presented promising data and prove its ease in the clinical workflow.

## Conclusions

In conclusion, ^166^Ho–^99m^Tc dual-isotope imaging allows automatic segmentation of the healthy liver tissue using a thresholding method without compromising assessment of healthy liver absorbed dose. ^166^Ho–^99m^Tc dual-isotope imaging paves the way for automated partition model-based activity calculation for ^166^Ho radioembolization, feasible in clinical practice. Prospective validation data are needed.

## Supplementary Information

**Additional file 1.** Bland–Altman plot and linear correlation between manual and automatic segmentation of the healthy liver VOI with respect to the D_70_ and V_50_.

## Data Availability

The datasets used and/or analyzed during the current study are available from the corresponding author on reasonable request.

## Abbreviations

^166^Ho: Holmium-166
^99m^Tc: Technetium-99m
CECT: Contrast enhanced CT
*D*_70_: Minimum dose to 70% of the volume
DVH: Dose–volume histogram
HD: Hausdorff distance
IQR: Interquartile range
LDCT: Low-dose CT
NET: Neuroendocrine tumors
REILD: Radioembolization-induced liver disease
SDC: Sørensen–Dice coefficient
*V*_50_: Volume receiving at least 50 Gy
VOI: Volume of interest
