# Supplementary material for: Automatic healthy liver segmentation for holmium-166 radioembolization dosimetry
Source: EJNMMI Res. 2023 Jul 15;13:68. doi: 10.1186/s13550-023-00996-1 (PMC10349793; doi:10.1186/s13550-023-00996-1)
Supplement: Supplementary file 1 — Additional file 1. Bland–Altman plot and linear correlation between manual and automatic segmentation of the healthy liver VOI with respect to the D70 and V50. [file 13550_2023_996_MOESM1_ESM.docx]

**Automatic healthy liver segmentation for holmium-166 radioembolization dosimetry**

Supplemental material


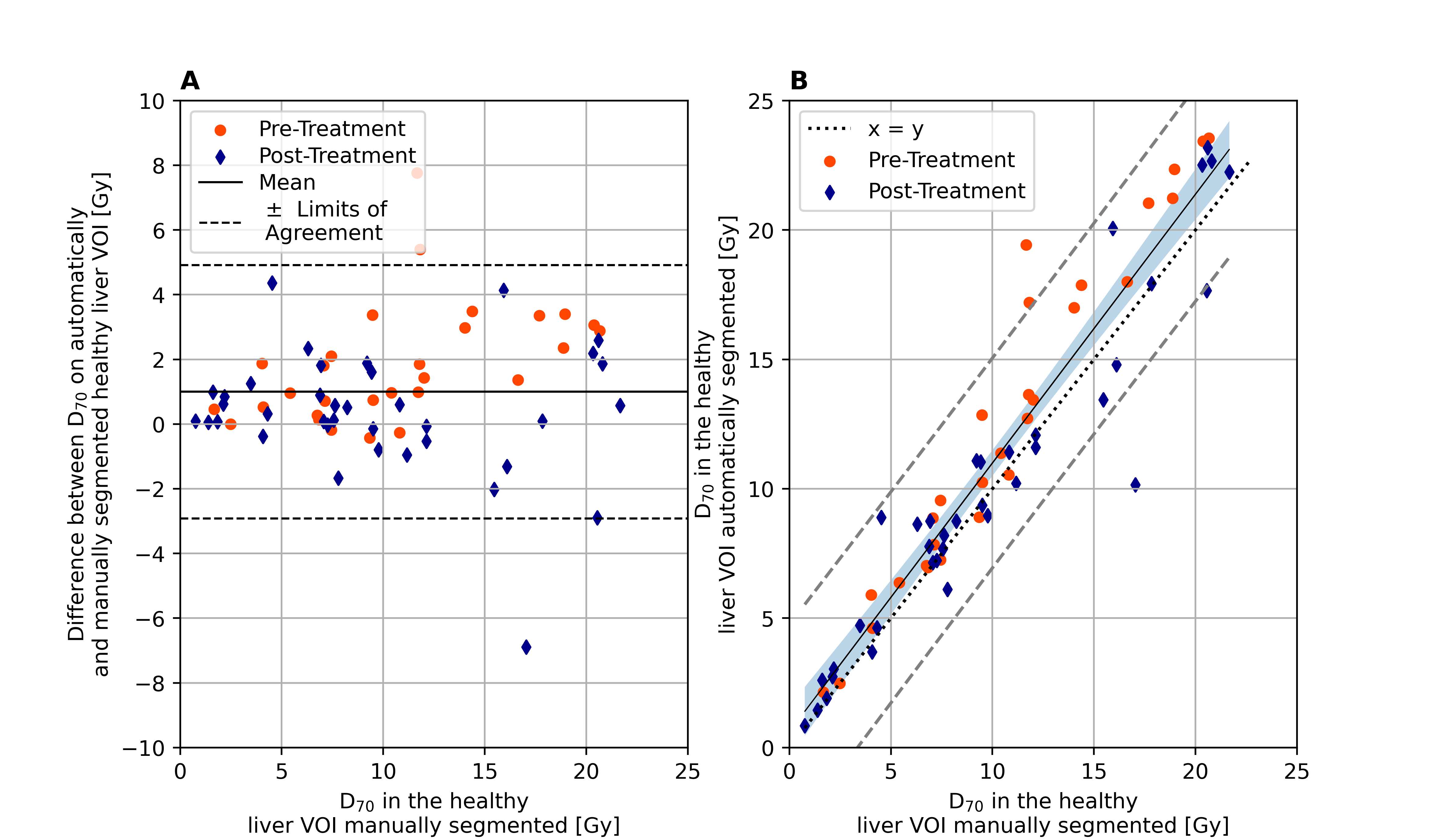


**S.1**

Panel **A**: Bland-Altman plot on difference between D_70_; D_70_ in the healthy liver VOI automatically and manually segmented against D_70_ in the healthy liver VOI manually segmented. Mean of difference is depicted by the black solid line, while black dashed lines define ± limits of agreement. Panel **B**: linear correlation plot between manual and automatic segmentation of the healthy liver VOI with respect to the D_70_. The solid line depicts linear regression, while the dashed lines indicate the ± 95% confidence intervals. Dotted line represents the x = y line. ^166^Ho pre-treatment images were scaled considering the therapeutic activity.


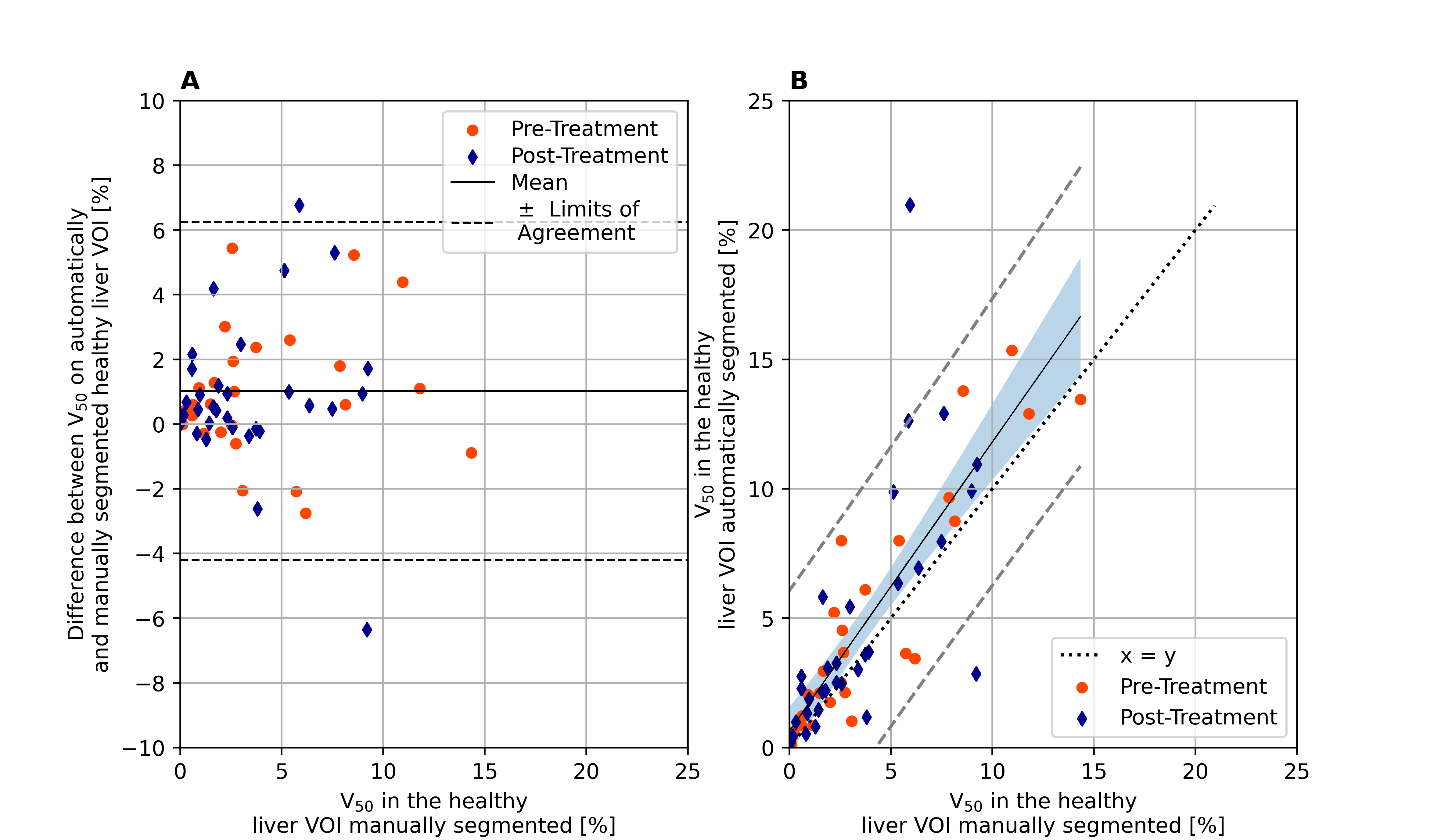


**S.2**

Panel **A**: Bland-Altman plot on difference between V_50_; V_50_ in the healthy liver VOI automatically and manually segmented against V_50_ in the healthy liver VOI manually segmented. Mean of difference is depicted by the black solid line, while black dashed lines define ± limits of agreement. Panel **B**: linear correlation plot between manual and automatic segmentation of the healthy liver VOI with respect to the V_50_. The solid line depicts linear regression, while the dashed lines indicate the ± 95% confidence intervals. Dotted line represents the x = y line. ^166^Ho pre-treatment images were scaled considering the therapeutic activity.
